# Partial epithelial–mesenchymal transition during enamel development

**DOI:** 10.1002/cre2.543

**Published:** 2022-02-19

**Authors:** Fayrouz Bazina, Sabine M. Brouxhon, Stephanos Kyrkanides

**Affiliations:** ^1^ Program in Oral Biology and Pathology, School of Dental Medicine Stony Brook University Stony Brook New York USA; ^2^ Translational Scientist, Center for Oral Health Research, College of Dentistry University of Kentucky Lexington Kentucky USA; ^3^ Department of Physiology, School of Medicine Stony Brook University Stony Brook New York USA; ^4^ Department of Oral Health Science, College of Dentistry University of Kentucky Lexington Kentucky USA

**Keywords:** ameloblast, hybrid phenotype, Oct4, partial EMT, Sox2, tooth development

## Abstract

**Objectives:**

We set out to investigate whether a hybrid stem‐like p‐EMT phenotype develops during murine molar enamel development in vivo.

**Setting and Sample Population:**

Histology specimens incorporating molar tooth buds harvested from mice at post‐natal day 4 (P4) were included in our studies.

**Materials and Methods:**

We employed double immunofluorescence staining to analyze the simultaneous expression of the epithelial marker E‐cadherin and the mesenchymal marker N‐cadherin in histology specimens with tooth buds harvested from P4 mice. Moreover, we evaluated the expression of the core master stem cell markers Oct4 and Sox2, as well as the mature ameloblast marker amelogenin.

**Results:**

Here we document the co‐expression of E‐cadherin and N‐cadherin in a sub‐population of pre‐ameloblasts in the inner enamel epithelium suggestive of the presence of a hybrid epithelial/mesenchymal phenotype resulting from p‐EMT. Moreover, the core stem cell factors Oct4 and Sox2 colocalized with E‐cadherin expressing pre‐ameloblasts, whereas the mesenchymal marker N‐cadherin was expressed specifically by amelogenin–positive mature secretory ameloblasts.

**Conclusions:**

The differentiation of E‐cadherin–positive pre‐ameloblasts towards N‐cadherin–positive mature secretory ameloblasts transition through a previously unidentified epithelial/mesenchymal stage derived through p‐EMT, co‐expressing the master transcription factors Oct4 and Sox2.

## 1 INTRODUCTION

Epithelial–mesenchymal transition (EMT) has traditionally been described as a developmental process in which cells undergo a complete epithelial (E) to mesenchymal (M) conversion (Kalluri & Winberg, 2009). However, recent research in cancer biology suggests the development of hybrid E/M cell states through a process termed partial (p)‐EMT (Aiello et al., 2018; Kalluri & Weinberg, 2009) and present with stem cell‐like markers (Chaffer & Weinberg, 2011; Chaffer et al., 2011, 2013; Kong et al., 2011; Valastyan & Weinberg, 2011). It is unclear, however, whether p‐EMT represents a state of transition between E and M phenotypes, or constitutes an end state per se (Aiello et al., 2018; Kalluri & Weinberg, 2009). Depending on the microenvironment, E cells may alter their phenotypical traits and display a combination of E and M traits (Kim et al., 2018). Accordingly, cancer cells in this hybrid E/M state can be identified by the co‐expression of E‐cadherin (E) and the M marker N‐cadherin (E/N hybrid), and simultaneously express stem‐cell associated factors that engender the tumor cells with varying clonogenic and differentiation potentials (Pastushenko et al., 2018). Among the stem‐cell factors, a member of the POU transcription factor family octamer‐binding protein 4 (Oct4), in complex with Sox2, is central to the machinery governing pluripotency and their joint expression has been shown to maintain the cancer stem cell‐like state (van Schaijik et al., 2018; Yu et al., 2007 *)*.

Previous reports in tooth development chart E‐ and N‐cadherin gradients within the developing enamel organ (Heymann et al., 2002). To this end, E‐cadherin expression in the dental epithelium followed an apical‐coronal gradient opposite to N‐cadherin during the cap and bell stages. Specifically, E‐cadherin was distributed in proliferating cells of the inner and outer enamel epithelia, whilst N‐cadherin expression was upregulated in differentiated cells, such as ameloblasts. Intriguingly, we recently reported on the development of a hybrid E/M cell phenotype starting with adult somatic oral keratinocytes treated chronically with TGFβ1 in vitro (Bazina, Brouxhon, & Kyrkanides, 2021). Moreover, we confirmed the plasticity of these cells through their differentiation into amelogenin‐secreting cells, and the subsequent production of a biomimetic enamel‐like material in vitro (Bazina, Brouxhon, Graham et al., 2021). However, we are unaware of whether p‐EMT and this stem‐like E/N hybrid exist in normal mammalian tooth development in vivo. Therefore, the goal of the present project was to interrogate whether a subpopulation of inner enamel E cells co‐express E‐ and N‐cadherin and the master stem cell factors Oct4 and Sox2, suggestive of a hybrid stem‐like E/M phenotype during enamel development in the mouse in vivo.

## 2 MATERIALS AND METHODS

### 2.1 Mouse tissue specimens

The heads of carcasses harvested from wild‐type C57BL6 mice (*N* = 3) killed at post‐natal day P4 were purchased from the Jackson Laboratory. The vendor has assurance in place for proper Internal Review Board approval for the breeding and maintenance of said mouse colony and the sacrifice of mice for research. The specimens were mailed by Jackson to us immersed in 10% formalin. Upon arrival to the laboratory, the specimens (heads) were paraffin‐embedded, cut into 5 µm histology sections, and collected onto glass slides.

### 2.2 Immunofluorescence

The aforementioned tissue sections were rinsed with phosphate‐buffered saline (PBS) and then blocked with 10% bovine serum albumin; antigens were detected by incubating with primary antibodies in PBS with 10% BSA and 0.4% Triton‐X overnight at 4°C. Primary antibodies used in this study were as follows: N‐cadherin (ab12221; 1:1000 dilution), Oct4 (ab18976; 1:1000 dilution), and amelogenin (ab153915; 1:1000 dilution) from Abcam; and E‐cadherin (sc‐7870; 1:1000 dilution) and Sox2 (sc‐20088; 1:1000 dilution) from Santa Cruz Biotechnology. Sections were rinsed in PBS and incubated in the following secondary antibodies: goat anti‐mouse Alexafluor‐594 (red) and goat anti‐rabbit Alexafluor‐488 (green) from Molecular Probes (Invitrogen). Cell nuclei were identified by the Hoechst counterstain (Invitrogen). The glass slides were cover‐slipped with ProLong^TM^ Antifade (Thermo Fisher Scientific, Cat# P7481) and fluorescent images were captured separately under 594 nm (red), 488 nm (green), and 350 nm (blue) wavelengths and processed using cellSens‐Standard software.

## 3 RESULTS

### 3.1 Inner enamel E cells express E‐cadherin

**Figure 1:**
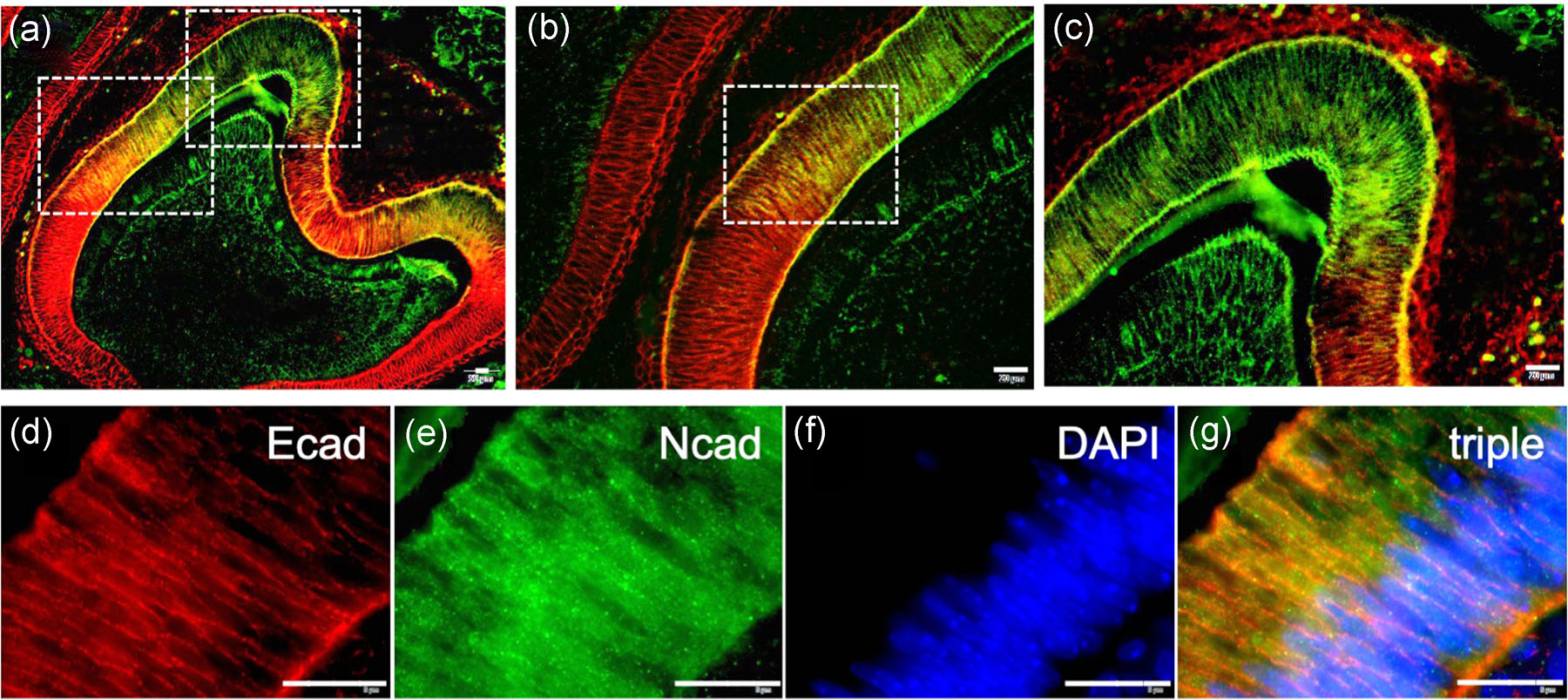
Co‐expression of E‐cadherin and N‐cadherin during enamel development E‐cadherin and N‐cadherin expression was detected by double immunofluorescence on histology sections containing tooth buds. (a) E‐cadherin (red fluorescence) and N‐cadherin (green fluorescence) coexpression (yellow fluorescence) was detected in a distinct region of the inner enamel epithelium, flanked apically by E‐cadherin (red fluorescence) and N‐cadherin (green fluorescence) on the opposite side. (b) Higher magnification of the enclosed area in (a). (c) Higher magnification of the enclosed area in (a) depicting the cusp region of the IEE. (d) Higher magnification of the enclosed area in (b) demonstrating E‐cadherin (red) immunofluorescence, (e) N‐cadherin (green), (f) DAPI immunofluorescence, and (g) triple fluorescence overlap of panels d–f. Bar = 50 µm. DAPI, 4′,6‐diamidino‐2‐phenylindole

As anticipated, due to their developmental origin from the oral epithelium, cells of the inner enamel epithelium for the most part express E‐cadherin, an E marker (Figure 1a). In this late bell stage, E‐cadherin is localized in cells apically to the region of the future cemento‐enamel junction (CEJ), including the cervical loop. All three specimens showed the same pattern of E‐cadherin expression.

### 3.2 Hybrid pre‐ameloblasts co‐express both E and M lineage markers during enamel development

Co‐expression of E‐ and N‐cadherin by a certain population of pre‐ameloblasts during the late cap stage tooth development indicates the development of a hybrid E/M cell phenotype. Here, E/M hybrid pre‐ameloblasts are located in the region of the future CEJ, and in the lateral walls of the central groove of the occlusal surface (Figure 1a–g). Close evaluation of this hybrid cell phenotype revealed pre‐ameloblasts whose nuclei are located basally, close to the basement membrane of the inner enamel epithelium (Figure 1d–g). This co‐expression of E‐cadherin and N‐cadherin by the same cell is a phenotypic characteristic of a hybrid phenotype developed through partial EMT. All 3 specimens showed the same pattern of E‐ and N‐cadherin expression.

### 3.3 Mature secretory ameloblasts undergo complete EMT

Mature secretory ameloblasts observed in the cusp region of the developing tooth, in contrast to the remainder of the inner enamel epithelium, are devoid of E‐cadherin (E marker), and solely express N‐cadherin (M marker) (Figure 1c), despite their E origin. The complete loss of E‐cadherin and the induction of N‐cadherin by this oral E‐derived cell type suggests that mature secretory ameloblasts have undergone complete EMT. All three specimens showed the same pattern of N‐cadherin expression.

### 3.4 Stem cell factors co‐express with E‐cadherin in pre‐ameloblasts

**Figure 2:**
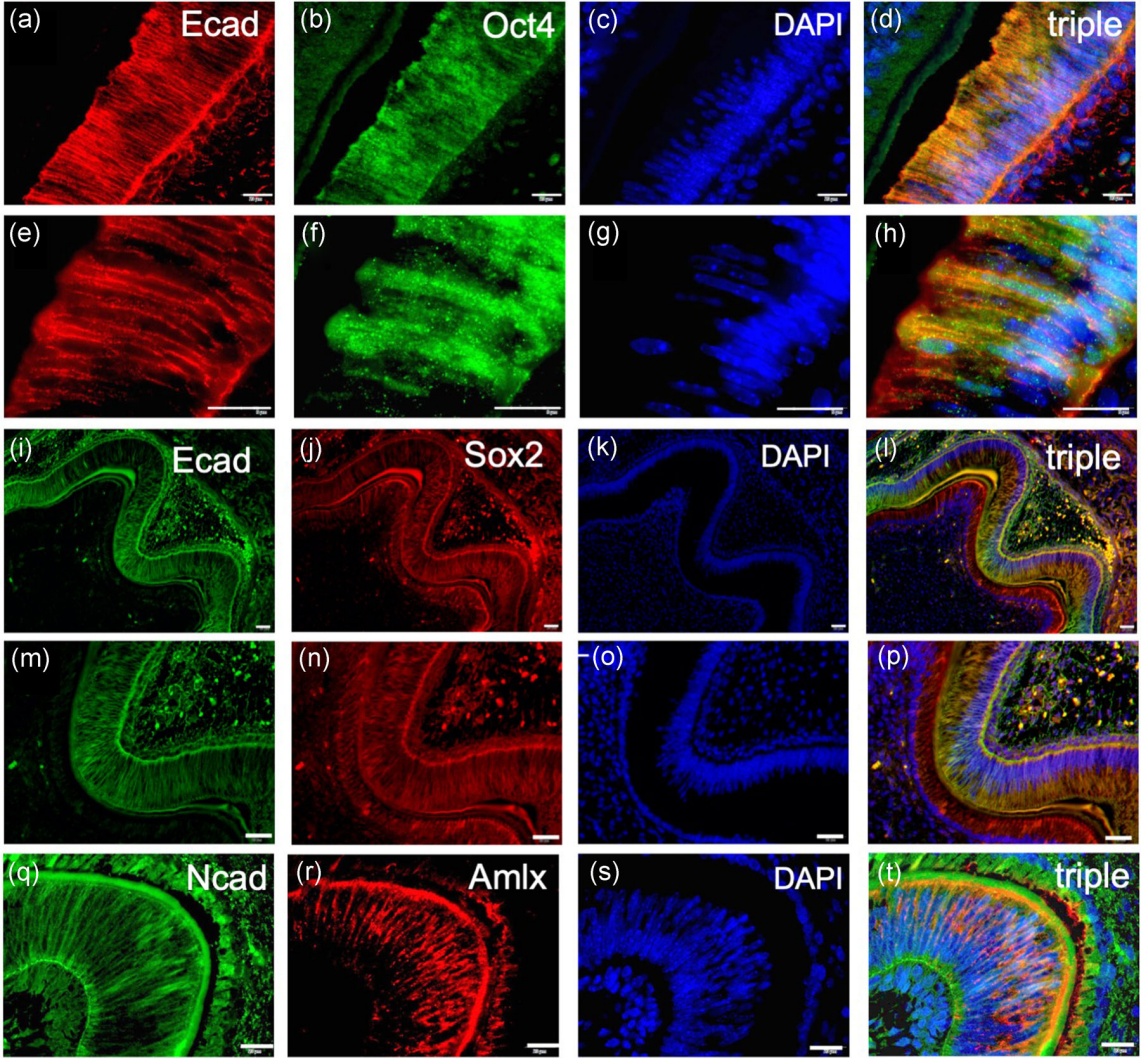
E‐cadherin expression is localized in pre‐ameloblasts, whereas N‐cadherin in secretory ameloblasts. (a) E‐cadherin (red fluorescence) and (b) Oct4 (green fluorescence) immunolocalized in pre‐ameloblasts within the inner enamel epithelium. (c) DAPI fluorescence was used to identify nuclei. (d) E‐cadherin colocalized with the stem cell factor Oct4 in pre‐ameloblasts. (e–h) are larger magnifications of the enclosures depicted in (a–d), respectively. Similarly (i–l) expression of E‐cadherin and of the stem cell factor Sox‐2 colocalized in pre‐ameloblasts. (m–p) are larger magnifications of the enclosures depicted in (a–d), respectively. Conversely (Q) N‐cadherin was immunolocalized in secretory ameloblasts within the cusp region, characterized by the expression of (R) amelogenin. (S) DAPI fluorescence. (T) Triple fluorescence overlap demonstrating colocalization of N‐Cadherin with the amelogenin in secretory ameloblasts. Bar = 50 µm. DAPI, 4′,6‐diamidino‐2‐phenylindole; Oct4, octamer‐binding protein 4

Expression of the transcription factors Oct4 and Sox2, which have been previously observed in pluripotent cells and associated with phenotypic stemness, were examined during tooth development. Interestingly, Oct4 expression was found to co‐localize with E‐cadherin in the inner enamel epithelium during the late bell stage of odontogenesis (Figure 2a–d,e–h). Similarly, Sox2 expression was observed to co‐localize with E‐cadherin within the inner enamel epithelium (Figure 2i–l), including the region of the lateral walls of the central groove (Figure 2m–p). All three specimens showed the same pattern of Oct4 and Sox2 expression.

### 3.5 N‐cadherin expression in secretory ameloblasts

The expression of the M marker N‐cadherin was identified specifically in ameloblasts co‐expressing amelogenin, a secreted keystone protein of the enamel matrix and marker of mature secretory ameloblasts, located in the region of the tooth cusp (Figure 2q–t). Interestingly, these are the cells that were found to be devoid of E‐cadherin (Figure 1a), showing that mature secretory ameloblasts selectively express N‐cadherin after complete EMT. All three specimens showed the same pattern of N‐cadherin expression.

## 4 DISCUSSION

EMT is a fundamental cellular process that plays a critical role in developmental pathways, tissue regeneration, and cancer. Recent research in cancer biology suggests that EMT exists as a continuum, whereby the cell proceeds through an intermediate state, termed partial (p)‐EMT, in which the cell harbors both E and M traits (E/M hybrid) and displays a high degree of cellular plasticity (Aiello et al., 2018; Kalluri & Weinberg, 2009). Herein, we set out to investigate whether this hybrid E/M phenotype develops during mammalian odontogenesis after partial EMT in vivo. Our results illustrate, for the first time, the presence of a subpopulation of pre‐ameloblasts co‐expressing E‐ and N‐cadherin, suggestive of the development of this hybrid E/M cell type during normal odontogenesis through p‐EMT. In support of our findings, we recently described the in vitro reprogramming of adult somatic oral keratinocytes into an amelogenin‐rich phenotype secreting a mineralized enamel matrix, that could be subsequently bioengineered to produce a scaffold‐free biomimetic enamel‐like material (Bazina, Brouxhon, Graham, et al., 2021; Bazina, Brouxhon, & Kyrkanides, 2021). Intriguingly, these reprogrammed somatic cells expressed both E‐ and N‐cadherin (unpublished observations), and exhibited stem‐like features (Bazina, Brouxhon, & Kyrkanides, 2021).

EMT is a highly dynamic cellular program in which polarized epithelial cells lose apical‐basal polarity, disassemble E cell–cell contacts and differentiate into a mesenchymal phenotype with enhanced cellular motility (Aiello et al., 2018). This cellular transdifferentiation from E to M states is mediated by key transcription factors, including Zeb, Twist, and Snail (Nieto et al., 2016). In contrast, p‐EMT is an alternative program that leads to the development of a hybrid E/M phenotype. Aiello and colleagues (2018) used a lineage‐traced tumor model to describe a p‐EMT program in several carcinomas that involved the intracellular re‐localization and retention of E proteins, such as E‐cadherin, rather than one involving transcriptional repression. This alternative EMT program led to a “partial EMT” phenotype that involved the formation of cell clusters that clinically formed tumor buds at the invasive front. Conversely, they found that complete EMT was associated with the transcriptional repression of E genes, including E‐cadherin (Aiello et al., 2018).

In the case of normal tooth development, hybrid E/M pre‐ameloblasts, located in the region of the future CEJ and central groove, transition from E‐cadherin–positive pre‐ameloblasts to N‐cadherin–positive secretory ameloblasts. Histologically, hybrid E/M pre‐ameloblasts that co‐express E‐ and N‐cadherin, flank the population of ameloblasts that will give rise to the cusps of the tooth. In parallel with the observations on tumor budding (Aiello et al., 2018), it would be tempting to speculate that the developing cusps may be a result of cell budding occurring from the regions of hybrid E/M pre‐ameloblasts through partial EMT. In contrast, mature secretory ameloblasts located in the tooth cusp region, as confirmed by the expression of amelogenin, selectively express N‐cadherin after complete EMT.

Pluripotent cells are a specialized population of cells that depend on transcription factors, such as Oct4, Sox2, and Nanog to maintain stemness, while simultaneously modulating lineage specification (Chakravarti et al., 2014; Chambers et al., 2007). Oct4 was the first master regulatory gene shown to be required for the induction and maintenance of pluripotency, and plays a critical role in the early stages of differentiation during embryonic development (Zeineddine et al., 2014). As such, Oct4 is well accepted as a pluripotency factor, in association with its partner transcription factor Sox2, both of which maintain a fine balance between pluripotency and their propensity to differentiate into a plethora of cells types that compose the three embryonic germ layers (Shi & Jin, 2010). Sox2 is known to maintain cells in an undifferentiated state and facilitate lineage commitment during embryonic development (Carrasco‐Garcia et al., 2016). Therefore, the E‐cadherin–positive population of inner enamel epithelium cells expressing Oct4 and Sox2 appear to be part of the pre‐ameloblast differentiation lineage towards mature secretory ameloblasts expressing both N‐cadherin and amelogenin.

In closing, the translational significance of our research is based on the fact that this previously unidentified E/M hybrid pre‐ameloblast may play a significant role in the development of tooth cusps during organogenesis. Moreover, this hybrid cell type developed as a result of p‐EMT may be critical in the bioengineering of biomimetic enamel materials for restorative dentistry. Future research experiments will be designed to elucidate the role of the hybrid pre‐ameloblasts during enamel development in vivo and the generation of enamel‐like biomaterials in vitro.

## CONFLICT OF INTERESTS

The authors declare that there are no conflict of interests.

## AUTHOR CONTRIBUTIONS

Fayrouz Bazina performed the bench top experiments. Sabine M. Brouxhon contributed to the experimental design and implementation of the experiments, identification of partial EMT, and composition of the manuscript. Stephanos Kyrkanides was responsible for the overall execution of the project and contributed to the experimental design, data interpretation, and manuscript composition.

## Data Availability

Data will be made available upon request.
